# Burning bright or burning out: a qualitative investigation of leader vitality

**DOI:** 10.3389/fpsyg.2023.1244089

**Published:** 2023-10-02

**Authors:** Jamie Shapiro

**Affiliations:** Claremont Graduate University, Claremont, CA, United States

**Keywords:** leader vitality, well-being, PERMA+4, emotional labor, emotional dissonance, self-control, energy transference

## Abstract

**Introduction:**

Leaders of organizations have incessant demands placed on them, including cultivating teams, building culture, and increasing the bottom line, in addition to caring for followers’ well-being and thriving. Numerous resources are required to meet these continuous demands, and vitality is one of the most valuable.

**Methods:**

Through interviewing 20 of the most influential and pressured leaders of *Fortune 1,000* companies, this qualitative study answers three important questions: what drains vitality, what fosters it, and how do leaders most effectively utilize vitality for followers?

**Results:**

The results shed light on psychological mechanisms that drain leaders’ vitality, including emotional labor, self-control, loss of job control, the unproductive mindsets of others, and isolation created from the role. In terms of fostering vitality, several of the pathways of the PERMA+4 model of well-being were highlighted, including fostering relationships, physical health, accomplishment, mindset, meaning, environment, and engagement. Two additional themes that foster vitality included job autonomy and time away from work. Themes emerged that underscore how leaders utilize their vitality for followers, and the potentially detrimental impacts to leadership when leaders are drained.

**Discussion:**

Overall, results highlight the importance of vitality and self-care as critical for leaders’ ability to maximize their leadership performance.

## Introduction

A recent survey of several thousand senior leaders across the United States found that 72% of leaders reported being burned out (HR Executive, 2021). Numerous resources are required to meet the continuous demands placed on leaders, and vitality is one of the most valuable. While many aspects of leadership have the potential to drain vitality, prior research suggests self-control, emotional labor, emotional dissonance, and energy transference may be particularly resource intensive. Understanding these mechanisms is an important first step in understanding some of the hidden costs of leadership. Exploring how and when these leadership mechanisms are enacted can provide additional insight into how to support a leader’s vitality, particularly for top leaders. Very few studies have focused on what drains and builds leader energy or energy transference to followers (Owens et al., 2016; Vogel, 2017; Cameron, 2021). Hearing directly from leaders about how and when self-control, emotional labor, emotional dissonance, and energy transference are enacted –and how these mechanisms affect vitality – is an important contribution to existing research.

Previous studies have shown that leaders are continually faced with enacting self-control in serving followers and putting the needs of others first (Liao et al., 2021). Self-control involves overriding or inhibiting behaviors, urges, emotions, or desires that could prohibit goal directed behavior (Muraven et al., 2006). Self-control can be excessively demanding for leaders due to the continual focus on suppressing their own desires in service of others, which can lead to mental fatigue, breakdowns in self-regulation, and drains in vitality (Liao et al., 2021).

Leaders also have requirements for emotional labor due to the need for leaders to always be “on” for their followers, conveying high levels of positive emotions and vitality to meet the demands of the role even when they are feeling depleted. Emotional labor is defined as the “effort, planning, and control needed to express organizationally desired emotion during interpersonal transaction” (Morris and Feldman, 1996, p. 987). The continuous need for emotional labor in effective leadership can come at a cost to vitality (Gardner et al., 2009). Understanding the direct experience of emotional labor from leaders who are required to be “on” for both internal and external stakeholders can shed new light into its impact on leaders.

Closely related to emotional labor is emotional dissonance, which is created when a leader portrays emotions that they do not feel. Emotional dissonance is defined as the conflict between the actual emotions experienced and organizationally required emotions (Middleton, 1989). Given that a leader’s emotions are contagious, leaders are continually faced with the need to portray emotions externally that they may not be feeling internally (Johnson, 2008; Banerjee and Srivastava, 2019). The disconnect between a leader’s internal emotional environment and external projection may cause leaders to feel inauthentic and disconnected from leading in accordance with their core values, identities, preferences, and emotions (Avolio et al., 2004). Leaders thus face a potential dilemma when striving for authenticity that can create emotional dissonance, drain vitality, and reduce well-being (Gardner et al., 2009).

Finally, at the heart of many leadership styles is the need for leaders to transfer positive relational energy to impact followers (Owens et al., 2016; Cameron, 2021). Positive relational energy is defined as an increased level of positive psychological capacities that are generated through interpersonal interaction, and leads to a person’s increased capacity to do work (Owens et al., 2016). Positive relational energy uniquely captures the actual transfer of energy resources in a leader-follower exchange (Owens et al., 2016), but it is also a mechanism that can drain leader vitality (Cameron, 2021). This study explores whether positive relational energy drains vitality in leaders, as well as its potential to renew energy.

This paper first reviews existing theory and research on vitality, followed by a review of the psychological mechanisms that drain vitality, and the antecedents of vitality. The qualitative study explores themes in leader vitality at the CEO level of *Fortune 1,000* companies. Methods and measurements are defined, and sampling and analysis strategy are reviewed. The results present the themes in what drains and fosters leader vitality, as well as, how vitality is utilized in leadership. The paper concludes with a discussion of the contributions of these potential findings, limitations, and suggestions for future research.

In sum, the current study explores the perspectives of leaders in high pressure, high visibility roles, to better understand what drains and fosters vitality and awareness. This study additionally sheds light on how leaders utilize their vitality for followers and the potentially detrimental impacts to leadership when leaders are drained. This study serves to enhance the current knowledge in vitality and advance the understanding of the importance of vitality in leadership. This understanding of leader vitality can help organizations support leaders, with the potential for positive long-term benefits for the organization.

## Vitality

The construct of vitality was first identified in 1997, when Ryan and Frederick (1997) defined vitality as positive aliveness and having access to the energy within oneself. However, vitality traces back to Ancient Greek and Eastern culture and philosophy in concepts like *Chi* in China, *Ki* in Japan, *Bayu* in Indonesia, and *Prana* in India (Lavrusheva, 2020). The common theme of these ancient concepts of vitality is an “underlying life energy or force flowing through living things” (Lavrusheva, 2020, p. 2). As stated above, vitality is an inner resource that includes physical energy, psychological energy, and emotional energy available to self (Shapiro and Donaldson, 2022). A key differentiator of vitality from energy is that vitality is associated with positively toned, energized states such as feelings of vigor (McNair, 1971), activated positive affect (Watson and Tellegen, 1985), and calm energy (Thayer, 1996) while the activation of energy is also associated with states like anger, anxiety, or arousal (Ryan and Frederick, 1997). More specifically, vitality is energy that one can harness for goal directed actions.

Due to its complexity, vitality continues to be a concept that is debated with little consensus on a single definition (Deng et al., 2015). In 2020, Lavrusheva reviewed 93 vitality studies, concluding that challenges in defining vitality arise because the construct is variously claimed to be an experience, feeling, or disposition with little consensus (Lavrusheva, 2020). The most agreed upon definition is that vitality is the energy available to self (Ryan and Deci, 2008); however, it is important to further define the components of that energy.

Vitality can be viewed as a higher order factor with three subfactors, including psychological, physical, and emotional energy (Shapiro and Donaldson, 2022). Most definitions of vitality are differing combinations of these three factors, leading to difficulty in applying the construct consistently (Richman et al., 2009). See Table 1 for an overview of the three vitality factors.

**Table 1:** Vitality factors.

Factor	Definition	Source
Psychological vitality	The mental energy available to oneself to think clearly, focus, be alert, have flexible thinking, and create a positive outlook	Richman et al. (2009)
Physical vitality	The energy available to oneself or a sense of aliveness in the body	Cannon (2011)
Emotional vitality	The energy resources available to oneself to regulate and express emotions effectively	Penninx et al. (1998)

### Leader vitality

Vitality is an important resource for both employees and leaders; however, the focus of this study is on leader vitality. A leader high in vitality experiences a sense of enthusiasm, aliveness, and energy (Ryan and Frederick, 1997). Vogel (2017) showed that when individuals feel energized and are thriving, leadership capacity is expanded. Vitality is an inner resource that is both generative and dynamic, and can help leaders cope with challenges, improve overall performance, and meet role demands (Dubreuil et al., 2014). Leaders can utilize vitality to manage or employ inner resources for intentional behaviors (Ryan and Deci, 2008).

**Figure 1:**
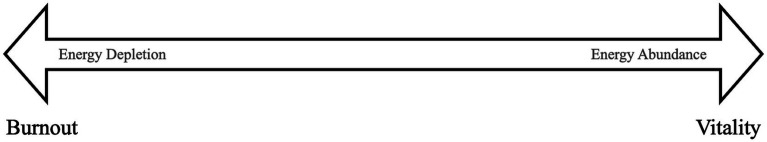
Burnout to vitality continuum.

Leaders increasingly need to serve a wide array of follower and organizational needs without depleting their own energy or risking burnout. Burnout is defined by Perlman and Hartman (1982) as “emotional/and or physical exhaustion, lowered work productivity, and over depersonalization” (Perlman and Hartman, 1982, p. 293). Vigor, defined by an abundance of energy and resiliency, has been shown to be the opposite of burnout (Bakker et al., 2014). Given that vitality has been characterized by energized states like feelings of vigor (McNair, 1971), burnout and vitality can also be seen as opposite ends of the energy spectrum (Figure 1). The definitions of both burnout and vitality include a physical component making vitality a more precise opposing concept to burnout, even more than engagement which is often seen as the opposite of burnout (Crawford et al., 2010). *Vitality can be defined as an abundance of physical, psychological, and emotional energy*, where burnout is a depletion of physical, psychological, and emotional energy. Given the incredible demands and expectations on leaders, vitality can be a path to burnout resilience.

Even though the definition of vitality has been fragmented, positive outcomes have been established. Vitality has been associated with both behavioral and objective health outcomes (Lavrusheva, 2020) including greater work performance, better coping with stress and challenge, more physical and psychological resilience, greater physical functioning and recovery, decreased anxiety, and heightened self-confidence (Ryan and Frederick, 1997; Penninx et al., 2000; Greenglass, 2006; Ryan and Deci, 2008). There are many positive antecedents of vitality including somatic health, well-being, physical activity, mental health, and work contexts including: meaningful work and successful work performance (Lavrusheva, 2020). Given the clear antecedents and benefits of vitality, leaders and organizations need a way to better understand what builds and drains vitality to protect leaders from depleting this important resource. An important first step is to understand the mechanisms needed in leadership that drain leader vitality, including self-control, emotional labor, emotional dissonance, and relational energy.

### Leader self-control

The changing world of business and public pressure has put a focus on more ethical leadership and business practices that improve the culture of the organization (Kiker et al., 2019). Putting follower needs first, like in servant leadership styles, requires self-control from leaders, and an orientation to serving followers has been associated with more ethical leadership behaviors, morality-centered self-reflection, good organizational decision-making, and less focus on power, fame, or self-centered goals (Giampetro-Meyer and Brown, 1998; Hoch et al., 2018). Self-control is defined as the “overriding or inhibiting of automatic, habitual, or innate behaviors, urges, emotions, or desires that would otherwise interfere with goal directed behavior” (Muraven et al., 2006, p. 524). Self-control helps leaders adapt to their social environment, be more responsive to others in their approach, have more interpersonal success, and focus on followers and organizational needs (Tangney et al., 2004; Baumeister and Alquist, 2009; Liao et al., 2021). However, self-control has also been shown to deplete energy resources and can lead to self-control fatigue (Baumeister and Alquist, 2009).

Leader self-control is needed in ever changing, complex environments, and increasing requirements for self-control can cause self-control fatigue and ultimately drain vitality. Multiple empirical studies have shown that increased levels of self-control can bring about lower levels of mental well-being and potential burnout (Diestel and Schmidt, 2009, 2011; Schmidt et al., 2007; Rivkin et al., 2015). Schmidt et al. (2007) showed through empirical evidence that self-control demands were significantly correlated with indicators of job strain and burnout.

In sum, engaging in self-controlling behaviors over time that focus so heavily on followers and the organization, while putting the leader’s needs second, can come at a vitality cost for leaders and potentially lead to burnout. Understanding the perspectives of leaders in some of the most demanding roles in the country can help uncover the potential costs of self-control on vitality.

### Emotional labor

Emotional labor is a continuous requirement for effective leadership in organizations and it can also be another drain on vitality (Gardner et al., 2009). Emotional labor is defined as the effort, control and planning needed to express emotions to others that are organizationally desired (Morris and Feldman, 1996). Leaders utilize emotional labor to alter their emotional expression in two different ways: (1) surface acting (when leaders change their outward emotional expressions but do not attempt to feel the emotions that they are displaying) and (2) deep level acting (when leaders attempt to feel the emotions they want to display) (Humphrey et al., 2008). Surface level acting has been shown to have the greatest negative impact to leader resources and well-being (Wang, 2011). An important contribution to the literature in emotional labor from this study is the deeper understanding of how top leaders handle the need for emotional labor in their roles.

Several research studies demonstrate that a leader’s emotions are contagious and can impact people both inside and outside of the organization (Johnson, 2008; Gardner et al., 2009; Banerjee and Srivastava, 2019; Li et al., 2019). Leaders have requirements to always be “on,” appearing with high levels of positive emotions and vitality – even when they are feeling depleted – in order to influence others toward organizational goals (Gardner et al., 2009). Many leaders do not have the permission to share when they are feeling depleted, burned out, or even languishing. Instead, leaders are expected to “show up” for their followers with the level of vitality and emotional regulation needed to inspire an organization toward a vision. This creates challenges for leaders to be truly authentic due to the requirements for continuous emotional labor.

Research on the impacts of leaders’ positive and negative emotions on followers has shown the importance of leader’s staying positive in the face of organizational demands (George and Bettenhausen, 1990; George, 1995; George, 2000; Lewis, 2000; Humphrey, 2002; Gaddis et al., 2004; Sy et al., 2005; Humphrey et al., 2008). Sy et al. (2005) provide evidence that a leader’s emotions are contagious. The leader’s positive (negative) moods induced group members to experience more positive (negative) moods and affective tone. Lewis (2000) similarly found that a leader’s negative emotions induced more follower negative affective states and less favorable assessments of leader effectiveness. A leader’s high levels of emotional labor can benefit followers and organizations, but the potential cost can be emotional exhaustion and drained vitality.

Emotional labor has been shown to detract from well-being, an antecedent of vitality, through emotional exhaustion and contribute to burnout (Erickson and Ritter, 2001; Brotheridge and Lee, 2002; Grandey, 2003; Glomb and Tews, 2004; Grandey et al., 2007; Johnson and Spector, 2007; Martínez-Iñigo et al., 2007). Emotional exhaustion is defined as a lack of energy and a feeling that one’s emotional resources are depleted. Awareness of emotional labor may highlight the hidden costs of leadership roles. In sum, emotional labor in the absence of opportunities for replenishment leads to emotional exhaustion and depletes leaders of the energy resources needed for vitality (Gardner et al., 2009).

### Emotional dissonance

While closely related, emotional dissonance is another potential drain to a leader’s emotional vitality. Emotional dissonance is defined as the conflict between the actual emotions experienced and organizationally or situationally required emotions (Middleton, 1989). Emotional dissonance is thus created when a leader portrays emotions that they do not feel which can happen in both surface and deep level acting. Emotional dissonance is a significant factor in emotional exhaustion, leading to lowered psychological well-being and acts as another drain to leader vitality (Van Dijk and Kirk Brown, 2006; Abubakar et al., 2022; Winkler et al., 2023). Emotional labor and emotional dissonance are directly linked with some conceptualizations that consider emotional dissonance as an antecedent to emotional labor (Zapf et al., 1999; Abubakar et al., 2022), and others position emotional dissonance as the labor component of emotional labor and a consequence of performing emotional labor (Rubin et al., 2005). However, emotional dissonance has been empirically shown to be an independent construct, distinct from emotional labor (Abraham, 1998, 1999; Van Dijk and Kirk Brown, 2006).

When leaders engage in either surface acting or deep acting, if the gap between the required self and the natural self is too large, it can lead to an internal state of tension which is emotional dissonance (O’Brien and Linehan, 2019; Abubakar et al., 2022). Erickson (1991) asserts that the fragmentation of the required self and natural self is harmful to the individual, and that over time this disconnect may lead to emotional exhaustion and job dissatisfaction. Emotional dissonance has also been found to be a key contributor to burnout (Kenworthy et al., 2014; Andela et al., 2016; Andela and Truchot, 2017).

Leaders often strive to be authentic by expressing transparency, openness, and trust, while at the same time remaining true to their inner thoughts, beliefs, and experiences (Gardner et al., 2005, 2009; Gardner and Carlson, 2015). Being authentic in leadership has been shown to have several positive impacts on employees, including empowerment, job performance, increased organizational citizen behaviors, organizational commitment, trust in leadership, and work engagement (Gardner et al., 2011). However, an important question that has been raised is whether leader authenticity can be attained while leaders are attempting to meet the emotional labor demands of their roles (Gardner et al., 2009).

When leaders are striving for authenticity but cannot be truly authentic in their emotions, it can lead to feelings of inauthenticity, creating emotional dissonance. While some research suggests that authenticity can contribute to leader well-being for leaders who can achieve authenticity, there is a question as to whether true authenticity is ever possible given the demands placed on leaders (Gardner et al., 2005; Ilies et al., 2005; Weiss et al., 2018). Hearing directly from leaders around this question contributes to the understanding of this complex issue. Weiss et al. (2018) showed that authenticity in leadership can build leader mental well-being through reducing stress and improving engagement. However, they also found that feelings of inauthenticity can deplete leaders’ mental resources, resulting in lower mental well-being. The emotional dissonance created from leaders striving for authenticity, and not being able to be truly authentic, can drain a leader’s vitality. Furthermore, when leaders are depleted, they cannot be authentic in their vitality, and therefore vitality becomes a potential negative loop for leaders because their desire for authenticity is not being met, creating even more emotional dissonance. High expectations for leaders are further compounded with the importance of leaders transferring positive energy to followers (Cameron, 2021).

### Energy transference – Positive relational energy

Prior research demonstrates that a leader can transfer energy resources to others through positive relationships which results in higher levels of engagement, lower turnover, and enhanced feelings of well-being among followers (Vogel, 2017; Cameron, 2021). The transference of positive energy from one person to another is called positive relational energy (Owens et al., 2016). Positive relational energy is defined as an increased level of positive psychological capacities that is generated through the interaction of people and leads to an increase in a person’s capacity to do work (Owens et al., 2016). Although vitality is positive energy, the crucial distinction is that vitality is an inner energy resource available to a leader, where positive relational energy is the transference of that energy to others. The continual need for transference of positive relational energy to followers thus creates another potential drain to leader vitality.

Cameron et al. (2011) explains that the transfer of positive relational energy creates a heliotropic effect which is defined as “the attraction of all living systems toward positive energy and away from negative energy, or toward that which is life giving and away from that which is life depleting” (Cameron et al., 2011, p. 288). The positive energy ripple effect is based on the theories and empirical research including positive energizers and energy networks theory (Cross et al., 2003; Cameron, 2021). According to Cameron (2021), some individuals are identified as positive energizers and create ripples of positive energy throughout the organization. There are substantial benefits of positive relational energy, but the outcomes have primarily been focused on the leader’s impact on followers and the organization, and there is limited literature and research on the required vitality needed from the leader.

For a leader to be a positive energizer in an organization and consistently transfer positive relational energy to followers, s/he needs a foundation of vitality or could risk burning out. Positive relational energy can potentially have a high energy resource demand from leaders without the vitality returns needed. A recent study showed that not all leaders feel energized from giving positive relational energy to followers, finding no direct relationship between positive relational energy and vitality (Shapiro and Donaldson, 2022). Cameron (2021) argues that positive relational energy is a renewable energy resource that energizes both the leader and the follower. However, it is possible that these energy relationships do not translate to leader-follower interactions due to power dynamics and the high expectations placed on leaders, making these relationships unique. This study explores these relational energy dynamics from the leader’s perspective to create greater clarity on how the mechanism of positive relational energy can potentially drain leader vitality.

### Fostering leader vitality

Given the drains in vitality that everyday leadership induces, it is important to further understand how a leader’s well-being can foster vitality and help protect against burnout. Lavrusheva’s (2020) literature review showed that there are many positive antecedents of vitality, and all are elements of well-being. Well-being is broadly defined as human flourishing and wellness of mind and body (Diener, 2009). Seligman (2018) developed the PERMA model for well-being that includes positive emotions, engagement, relationships, meaning, and accomplishment. Donaldson et al. (2020) added four additional elements to the PERMA model: physical health, mindset, environment, and economic security. PERMA+4 has been validated through extensive research and measurement and has been shown to have a strong link to work role performance (see Table 2).

**Table 2:** Elements of PERMA+4.

PERMA + 4	Definition
Positive emotions	Experiencing happiness, joy, gratitude, etc.
Engagement	Using your strengths to meet challenges; experiencing flow
Relationships	Connecting with others; love and be loved
Meaning	Connect to meaning; find your purpose
Accomplishment	Pursue and accomplish goals; strive for greatness
Physical health	Biological, functional, and psychological health assets
Mindset	Future-oriented, growth mindset, perseverance
Environment	Spatiotemporal elements, such as access to natural light, nature, physiological safety
Economic security	Perception of financial security

Donaldson et al. (2020).

Antecedents of vitality can be classified into psychological, physiological dispositions, and external circumstances or life events (Lavrusheva, 2020). The psychological antecedents can all be classified as either well-being or mental health factors, including both positive meaning and emotions. Physiological antecedents include somatic factors of well-being (Lavrusheva, 2020). The PERMA+4 model of well-being most closely aligns with the antecedents of vitality and has been shown to build vitality in leaders (Shapiro and Donaldson, 2022). Cultivating positive emotions and a positive mindset has been shown to help minimize the well-being costs of emotional labor (Humphrey et al., 2015). Relational support, another key element of PERMA+4, has also been shown to have a buffering effect against the negative effects of emotional dissonance (Abraham, 1999). Meaning in life has also been shown to be an important antecedent to vitality even when people are unable to engage in regular physical activity (Ju, 2017).

Weiss et al. (2018) showed that authentic leadership can improve leaders’ mental well-being through reducing stress and increasing work engagement when leaders can be truly authentic in interactions with followers. The Weiss et al. (2018) study underscores the importance of bringing into alignment leader vitality and the energy they are sharing with others, highlighting the leader-follower relationship. Aligning the internal and external worlds of leaders allows more authenticity in their interactions. Perhaps this alignment for leaders is the missing link for creating reciprocal positive relational energy that builds leader vitality through follower interactions. This qualitative study explores how leaders build vitality, and how follower interactions can potentially enhance leader vitality. This study investigates how vitality is fostered and drained at the highest levels of leadership.

## Methods

Using qualitative methods with semi-structured interviews, this study illuminates the themes in leader vitality at the CEO level of *Fortune 1,000* companies. The purpose of the phenomenological study (an approach that seeks to understand the essence that all persons experience about a phenomenon) was to understand the concept of leader vitality through the experiences of CEOs in large scale organizations, gaining a list of the factors that both drain and foster leader vitality (Creswell et al., 2007). The phenomenology approach allows a deeper understanding of CEO experiences and exploration of the subjective aspects of vitality, providing valuable insights. Leveraging existing research and knowledge, this study seeks to validate known factors and explore additional elements not previously identified. Additionally, a key contribution is the understanding of how leaders utilize vitality to impact their followers. Exploring through the lens of some of the most influential and high-pressured leaders provides clarity into leader vitality. As Rod Laird Organisation (2012) argues, extreme sampling includes unusual or extreme conditions that are relevant to improving other, more typical situations.

### Research questions

The research questions that guided this study are:

1. What drains leaders’ vitality?
2. What fosters leaders’ vitality?
3. How do leaders effectively utilize their vitality for their followers?

### Participants

This study includes *Fortune 1,000* CEOs or equivalent, who face extreme demands both internally and externally as well as high expectations from multiple stakeholders. These CEOs must report to their followers, the Board, shareholders, and investor communities. They are required to be “on” continually with high levels of vitality to tackle strategy, vision, organizational culture, quarterly earnings, and the survival of the organization. The sampling criteria included a Board reporting structure, internal and external stakeholders, over 4,000 employees, at least six executive level direct reports, and financial responsibility for over US$1 billion.

Based on the similarities in pressures, demands and responsibilities of the CEOs that were selected for this study, the sample size included a total of 20 participants. Theoretical saturation (the point in data collection when no additional insights are identified about the theoretical construct) was used to determine if the sample size was sufficient, and that point was reached at 12 interviews. However, 8 additional interviews were conducted as a further step in validation (Hennink and Kaiser, 2022).

One of the largest challenges of the sample was the homogeneity of this population and lack of diversity; almost 90% of *Fortune 500* CEOs are white males (The Society Pages, 2020). There is not only a lack of gender diversity and homogeneity in race in this population, but also ethnicity, functional and educational backgrounds, age, and socioeconomic status. Participants recruited for this study were a convenience sample from the researcher’s network; however, additional efforts were taken to recruit as diverse a sample as possible (see Table 3).

**Table 3:** Participant demographics.

Participant	Demographic information	Frequency
Gender		
Male		16
Female		4
Ethnicity		
White or Caucasian		15
Black or African American		1
Hispanic		2
Middle Eastern		1
Asian		1

To recruit participants, email requests were made for a 30-min interview. Participants did not receive any compensation for their participation; however, they did receive a “thank you” gift in appreciation for their time. Interviews were audio recorded using Zoom software, transcribed using Otter.ai software, and identifying information was removed prior to analysis using MAXQDA. Interviews were recorded for transcription purposes only and deleted after transcription. All names have been changed to pseudonyms, and all identifying information has been removed from the quotations below.

### Measures

The semi-structured interview protocol can be found in Table 4.

**Table 4:** Interview protocol.

Section heading	Sample question
Opening	Define vitality for participants – vitality is defined as positive aliveness and having access to the energy within oneself including emotional, psychological, and physical energy.
Understanding what drains vitality	Think about a day when you felt emotionally, psychologically, and physically depleted of energy. Can you walk me through the details of that day? Potential follow up questions: What interactions did you find the most draining? What aspects of your role where most depleting? What did you notice about your environment that day? What was different about that day from others? What do you notice about yourself that day? What behaviors did your team notice? Do you think others knew you were depleted? Why or why not?
Understanding what fosters vitality	Think about a day when you felt emotionally, psychologically, and physically energized, full of vitality. Can you walk me through the details of that day? Potential follow up questions: What interactions did you find the most energizing? What aspects of your role where most energizing? What did you notice about your environment that day? What was different about that day from others? Where there any practices/routines that contributed to you feeling vital? How did you utilize your vitality that day? What behaviors did your team notice? Do you think others knew you were feeling vital? Why or why not?
Closing questions	Is there anything I did not ask, but you think I should have?

### Thematic analysis

Thematic analysis was utilized, a common approach when existing theory is limited (Hsieh and Shannon, 2005). Data was systematically sorted, summarized, and compared while identifying the major themes around leader vitality. It was important to use a flexible approach in the face of emergent findings, as well as grounded in prior research. Given prior research in leader vitality, an *a priori* coding scheme was utilized and combined with inductive codes that emerged. Table 5 details the initial coding scheme.

**Table 5:** *A Priori* coding scheme.

Leader vitality	Code description
Antecedents – Foster Based on the PERMA+4 Model	Positive emotions Engagement Positive relationships Meaning Accomplishment Health Mindset Environment Economic security
Drains – based on psychological mechanisms	Self-control Emotional labor Emotional dissonance Energy transference

As recommended by Kondracki and Wellman (2002), interview transcripts were initially reviewed in entirety to gain a holistic understanding and capture key concepts. Next, initial impressions and *a priori* codes were applied. The data was subsequently analyzed for emergent themes not initially identified in the coding scheme, data was then organized into broader categories of themes and a final set of codes was identified (see Appendix A in Supplementary material). This approach led to findings that are grounded in the data of each participant’s unique perspectives (Hsieh and Shannon, 2005).

### Enhancing validity

Qualitative research allows for rich contextual insights into CEO’s lived experiences in relation to vitality, but it was essential to eliminate as many threats to validity as possible. Given the self-report nature of the semi-structured interview format, the steps outlined by Creswell and Poth (2018) were taken for the interview protocol. Questions were designed to be open ended, short, and clear.

Another threat to validity was related to the recruitment process. Many of the CEOs interviewed are clients that work directly with the researcher and therefore have existing knowledge related to what fosters leader vitality. To minimize this threat, 8 of the 20 CEOs recruited had no previous experience with the researcher. Data was also collected past the point of saturation to minimize this potential validity threat.

A final area of concern was researcher bias and prior experience in the topic of leader vitality. To minimize this threat, all interviews were recorded and transcribed for analysis. This minimized the tendency to only capture data that fit existing theory, goals, or preconceptions (Maxwell, 2004). In addition, through the process of transcription, each full interview was reviewed prior to analysis.

## Findings

The findings of this study are divided into three sections. The first two sections explore what drains versus fosters leader vitality, delineated by expected results and emergent themes. The third section describes leaders’ uses of vitality for followers.

### What drains leader vitality

As expected, many of the psychological mechanisms outlined in prior research were identified as drains in leader vitality. There were also three emergent themes – loss of job control, the unproductive mindsets of others, and isolation created from the role.

#### Expected themes – drains to leader vitality

The psychological mechanisms of emotional labor, self-control, energy transference, and emotional dissonance were all identified by participants (refer to Table 6).

**Table 6:** Drains to leader vitality – psychological mechanisms.

Theme	Definition	Example quote	Participant count
Drains to leader vitality			
Emotional labor	The effort, control and planning needed to express emotions to others that are organizationally desired	I put up a good front, I think the shadow we cast as leaders is important. You cannot have a bad day. (P17)	14
Self-control	The overriding or inhibiting of automatic behaviors, urges, emotions that interfere with goal directed behavior	Somebody will be presenting something and you are like, I really do not even understand why you are talking to me about this. But you cannot say that, you have to use self-control in those moments. (P3)	9
Energy transference	The transference of positive energy from one person to another	It’s how I can actually push all that vitality and energy into others, which slowly drains me. (P15)	4
Emotional dissonance	The conflict between the actual emotions experienced and organizationally or situationally required emotions	(In reference to being drained) I want a persona of approach and availability. And I use the term I like To be anti-corporate, even though we have got thousands of employees, I still want it to be a family style culture. So that piece is very important to me and I will do my best to fake, and be a pretender to a degree. (P14)	3

##### Emotional labor

Seventy percent of CEOs were aware of emotional labor demands and the consequential vitality drains. Participant (P10) explained “Had I shown everything that I was feeling, they would have just seen me super, super mad. That’s how it would have showed up. So that’s what I am fighting off.” Several leaders shared their use of emotional labor to prevent negative impacts to others from their internal negative emotions.

##### Emotional dissonance

Very few (15%) participants pointed out the emotional dissonance created from the disconnect between their internal and external feelings. One participant (P17) shared that he had become more authentic in his leadership. He explained that he used to believe leaders could not express negative emotions, but now he feels, “To be an authentic leader, you have to be real, acknowledge sometimes that you are not up to par.” He shared that his authenticity has created more opportunities for connection and engagement with his team. His insight aligns with Weiss et al. (2018), who showed that authentic leadership can improve leaders’ work engagement.

##### Self-control

Another mechanism that almost 50% of the leaders cited was self-control. A leader (P9) aware of the importance of withholding information explained, “I cannot necessarily bring that information to my team, because it can create a little bit of uncertainty or loss of confidence, potentially, depending on the topic.” There were several examples given about the need to adjust leadership styles based on the audience.

##### Energy transference

Only 20% of participants discussed energy transference as a drain to vitality, although the number one theme in uses of vitality (65% of leaders) was the transfer of positive relational energy (see Leadership Behaviors section below). One leader (P8) drained from energy transference shared:

We have an annual leadership festival. We get everybody together, all our leaders, and it’s usually two and a half days, lots of interactions with people. And I always find myself the next morning, to be spent of energy. I am just ready to just kind of escape, I’ve had two and a half days of having to be on nonstop.

Cameron’s research Cameron (2021) shows that positive relational energy can be a renewable resource that energizes both the leader and the follower. However, this study’s findings are consistent with previous research that positive relational energy does not always positively impact the leader (Shapiro and Donaldson, 2022).

#### Emergent themes – drains to leader vitality

Three other main themes, which are detailed in Table 7, emerged as drains to leader vitality: loss of job control, unproductive mindsets of others, and the sense of isolation inherent in the role.

**Table 7:** Drains to leader vitality – additional themes.

Theme	Definition	Example Quote	Participant Count
Drains to Leader Vitality			
Loss of Job Control	Overall feeling of not being in control of the job including the calendar (back-to-back meetings), disruptions caused by unforced errors, low value task and meetings, and external factors causing negative impacts to the workforce	It’s disruption that seems unnecessary, misdirected. And it slows me down. Somebody else’s lack of preparation, or progress or somebody else’s idea of what is urgent, or an emergency. That throws me off, my current cadence, it sucks a lot of energy out of me. (P4)	17
Mindsets of others – negative, fixed, self-oriented	People that are self-oriented, have a fixed mindset or overly focused on the past. This includes team members, the Board, analysts, and employees. These mindsets erode trust	The promise – you swear to tell the truth, the whole truth, and nothing but the truth. I tell people all the time, too many people in corporate America, forget that middle bucket, the whole truth. They know, they know exactly what you are asking them, they know what you are driving towards. And they answer the piece that gets them hopefully the outcome they want. (P15)	13
Isolation	The isolation of the role and lack of people to honestly talk to. Working from home created another factor in feeling alone	It was so stressful, not having any colleagues that I felt like I was part of a team with other than my direct reports. And so there was really no one to talk to. I went from having three colleagues that were in the same line as I was to not really having anyone. (P11)	6

##### Loss of job control

A key theme highlighted was loss of job control, with the most mentioned area being loss of calendar control due to back-to-back meetings, low value meetings, and unexpected urgent issues. Leader (P16) explained:

I get done with a meeting and I have my admin knocking on the door because something else has come up and I’ve literally got a minute to get to the next meeting. Then I’m walking down the hall and there’s the person grabbing me because I’ve been in meetings all day. You’re giving so much of yourself, and you’re still being pulled on.

Another participant (P3) stated when he felt most depleted, “It’s a day where I feel as though I’ve lost control of the calendar, and how my time is being spent.”

These findings are consistent with research around job control, which is defined as a broad concept referring to the degree to which people can choose their actions (Spector, 1998). Lack of control was identified when leaders had to direct time and energy to low value, low impact tasks. One leader (P15) shared, “But then add into it the typical things that happen in a CEO job, which is in the middle of the day, you get these, I would argue, inconsequential problems, [that] come to the table [because] people push them up.” Leaders also expressed how unforced errors, especially by the executive team, led to a significant drain in vitality. “I do not like when we make unforced errors, and it feels like the reason I’m paying attention to this thing and not what’s more important to the organization is because of something we did to ourselves” (P2). The final factor in the job control theme was external circumstances out of the leader’s control that caused negative impacts to the workforce, including the pandemic. A leader (P8) shared, “I recall making that decision (to lay off employees). I literally cried in our boardroom with our executive team.”

##### Counterproductive mindsets of others

Another theme that emerged was the impact of counterproductive mindsets including team members, the Board, analysts, and employees. Sixty-five percent of leaders shared that they became drained of vitality due to the unproductive mindsets of others. These draining mindsets include: (1) a focus on self, versus the organization [“It’s people doing things for reasons related to their own agenda or their own interest rather than the good of the organization.” (P2)]; (2) people with a fixed mindset or a mindset overly oriented toward the past [“I do have those days where people tend to cling to what they know, as opposed to, bring in their mindset of learning. And that’s frustrating for me.” (P7)]; and (3) negative mindsets [“I hate negative energy; it just really drains me. When someone is like, this sucks, and that sucks or they are manipulative or you cannot trust them, trust is another big one. If I’m surrounded by stuff that I cannot trust, or I’m not sure, I do not believe it, that starts to drain my energy” (P5)]. Three participants expressed the erosion of trust as a drain to vitality due to people’s negative mindset and self-orientation which made leaders feel that others were being dishonest and manipulative. This finding is consistent Dutton (2003) which explains that low quality connections take an energetic toll on people, leaving them feeling depleted.

##### Isolation

The final theme that emerged was the isolation of the CEO role. Leader (P17) shared, “It’s lonely sometimes, I could call somebody, but everybody’s busy.” The feelings of isolation have only increased with working from home. A participant (P5) explained:

When I’m in the office, it’s give and take; I give energy and I get replenished from some other conversation happening here. Whereas when I’m working from home, I’m just like giving, giving, giving, giving, and then by the end of the day, I’m just dead.

The results of this study illustrate a list of the drains to leader vitality from both the validation of previous research and additional insights gained. Equally important is the understanding of what fosters vitality, the topic of the next set of findings.

#### What fosters leader vitality

Overall, the PERMA+4, as a model for fostering leader vitality, was largely supported. Additional themes emerged including the importance of creating thinking space in the day and time away from work.

#### Expected themes – Fosters of leader vitality

As expected, the PERMA+4 is a key to fostering vitality with 100% of participants citing one or more pathways of the PERMA+4 model of well-being. This finding is supported by Lavrusheva’s (2020) literature review which showed that the positive antecedents of vitality were all are elements of well-being. One leader (P18) aware of the importance of well-being explained, “Healthy heart, healthy mind, healthy body all goes along.” Relationships were the most mentioned theme followed by physical health, accomplishment, mindset, meaning, environment, and engagement. See Table 8 for a summary of the themes that were mentioned 5 or more times. Positive emotion was only mentioned by one participant and economic security was not mentioned at all in the interviews. Given this population’s socioeconomic bracket, it is not surprising that economic security was not a theme. However, it was unexpected that this sample did not mention positive emotions since previous research and theory has shown that positive emotions foster vitality (Lavrusheva, 2020).

**Table 8:** Fosters of Leader Vitality – PERMA+4.

Theme	Definition	Example quote	Participant count
Fosters of Leader Vitality – PERMA+4			
Relationships	Connection with others: family, friends, significant other, and work relationships	My energy is at its peak when I’m in person with a group of my colleagues, team, or clients. (P6)	20
Health	Biological, functional, and psychological health assets	I would exercise and drink a smoothie and felt I started this thing out the right way today, like I already accomplished something first thing in the morning. And that really did bring energy and vitality. And it felt like I started off my day as a winner. (P1)	17
Accomplishment	Pursuing and accomplishing goals; striving for excellence	I get a lot of energy, when, you know, a well laid out plan comes together. (P4)	13
Mindset	Growth mindset, resiliency, perseverance, positive outlook	I have people who are high in optimism, competence and conviction around dealing with an issue, and we all feel that way, and we work together, I feel great. I just I feel like I’m on top of the world, I get more energy out of that. (P5)	10
Meaning	Connection to meaning, purpose, and impact	I deeply believe in the services that we provide to our fellow citizens. And so, when I see our company delivering those important services, that’s what keeps me going every day, because it’s so important. Beyond the business side, it’s so important just to be able to help people in the way that we can. And so that’s where I get a lot of a lot of satisfaction and energy. (P13)	9
Environment	All environmental factors related to life and work including, access to natural light, nature, physiological safety	Environmentally, just being here or any of our offices or any of our client offices, that’s a big stimulant, is being among people. (P6)	8
Engagement	Using strengths to meet challenges, experiencing flow	I’m talking to people about strategic initiatives, or new things, or some kind of growth that we are doing is much more energizing. (P2)	5

##### Relationships

A key theme is positive relationships, both personally and professionally, matter when it comes to fostering vitality. Several leaders shared increases in vitality from relationships with family and friends, “I’m able to maintain bonds, with close friends and family, like my son and my wife, and just feeling those bonds is another thing that I think is just something I need” (P5). Another leader (P2) shared that he feels most vital when “I’m feeling particularly connected to my family.”

Professionally, 50% of leaders discussed the vitality they gained from their teams, particularly in helping them develop. One leader (P3) expressed that he felt vital when he had the “opportunity to coach people on the team and help them become bigger and help them grow their own capacity and capability.”

Another significant theme was the time spent with frontline employees. One CEO (P9) captured the exchange of the energy he felt:

Number one, I feed off of their energy, when they talk about how passionate they are about the work that we’re doing. When they clearly appreciate the fact that their CEO is spending time and engaged and asking questions. It’s so validating, this mutual energy that’s going back and forth.

The quality of relationships was also discussed as a contributor to vitality with six leaders mentioning trusted relationships, such as P8’s comment: “The closeness to my team, just in terms of, really great, open conversations. I have no boundaries, no barriers to having this really open dialogue.”

##### Physical health

Physical health was discussed by 85% of participants as a critical element in fostering vitality; from exercise and movement to nutrition to sleep, physical health was discussed repeatedly. “Doing some kind of physical activity gives me great energy, not necessarily in the moment of doing it, but afterwards” (P9). “I sleep so it’s probably the one thing I do. I go to bed early, and I try to always get 8 h” (P7). “You know [I am most vital when], my diet is good. And getting appropriate amounts of rest” (P13). Many leaders also discussed the importance of meditation, “I will take time to do my Calm app” (P17). “I have to find a way to release, which I do at times when I do start to sit down and meditate” (P3). This finding is in alignment with previous research that shows somatic health including physical activity, healthy lifestyle habits, and good sleep are all antecedents of vitality (Lavrusheva, 2020).

##### Accomplishment

Another clear vitality booster for 65% of the sample was accomplishment. Many leaders discussed that a day in which they are high in vitality is a day where they feel like they are “winning.” As one leader (P13) expressed, “I do get fulfillment when we win at something.” Another leader (P14) shared it is a day when, “I get to celebrate something. I can celebrate the accomplishment of one of our leaders or in our business metric that we hit.”

##### Mindset

Half of the leaders discussed the importance of a productive mindset and pointed to the elements of psychological capital including hope, efficacy, resiliency, and optimism. A leader (P5) explained “A can-do attitude, optimism, competence, conviction, those things are critical to me.” The same leader pointed to the importance of self-efficacy, “Confidence is so critical to my levels of vitality.” Another leader (P11) stated very clearly, “Your mindset matters in building your vitality.” Leaders also discussed the importance of a growth mindset, “And so for me a big part of vitality for me personally, is I’ve realized that what has been my fuel my whole life has been the newness of things, the uniqueness of things” (P15). Not only was the leader’s mindset a direct contribution to vitality, but also the positive mindsets of those around them. Leaders gain vitality from people with positive or growth mindsets. A participant (P5) shared, “There are certain people that are a source of energy for me that help me and it’s because they always have that energy, or are super enthusiastic, happy, confident and optimistic.”

##### Meaning

Connection to impact, meaning and purpose were mentioned by almost half of the leaders. One leader (P15) expressed:

It will be the people that have worked with me or for me: I feel a real sense of joy and excitement and accomplishment when that individual reaches out and says something like, you may not remember me, but I was at this meeting and it really impacted my career, or it really impacted my home life, or it really impacted me.

Another leader (P17) directly pointed to purpose, “So that’s the piece that has really helped me, living on purpose or purpose driven living. Touching people’s lives gives me energy but also gives them energy as well.” The connection between meaning and vitality has been validated by previous research showing that meaning in life fosters vitality in elderly adults (Ju, 2017) and another study showing the same in nursing populations (Lee and Oh, 2007).

##### Environment

Forty percent of leaders spoke about how their environment impacts their vitality. “Our headquarters, just the colors and the people working and collaboration and walking through the hallways, it fills my cup up, to stop in the hallway” (P5). “Environmentally, just being here or any of our offices or any of our client offices, but that’s a big stimulant, is being among people” (P6). “I have my highest level of vitality when I’m when I’m actually in an office interacting with and able to collaborate quickly, able to ask questions quickly” (P10). Of this subset of 8 leaders, 5 talked about the weather contributing to their vitality, e.g.: “I always feel better in sunny weather” (P2).

##### Engagement

The final theme of the PERMA+4 that emerged was engagement. Only one quarter of the leaders discussed engagement as a source of vitality; however, over 50% of leaders discussed engagement as an outcome of vitality. It appears from this study that engagement is both an antecedent and an outcome, with more awareness around engagement as a consequence. The greatest recognition of engagement as an antecedent centered around strategy, “I find big strategic movements, energizing, making a deal,” (P2) and another participant (P3) spoke about a sense of flow with the team, “feeling that the team is learning that you are moving forward that things are clicking.” Two participants talked about the vitality gained from utilizing their strengths including focusing on energizing tasks, “I choose to pay attention to the ones (tasks) that give me energy and ignore the ones that suck energy” (P6).

#### Emergent themes – Fosters of leader vitality

Two other themes emerged: (1) job control in terms of space for thinking, and (2) non-work time, which included any time away from work. Refer to Table 9 for an overview of these themes.

**Table 9:** Fosters of leader vitality.

Theme	Definition	Example quote	Participant count
*Fosters of leader vitality*			
Job Control – Creating Space for Thinking, Being Organized, Prepared	Time and space for thinking that is not back-to-back meetings. Time to get organized and feel prepared	I can schedule my day to stay outside of the troughs and understand what energy I’m going to need. Those are some of the best days. The other thing, no surprise, are the days where there is me time. Yeah, it is when there is space, it’s when I wake up in the morning and I have allowed myself and scheduled the 45 Minute do “me time.” (P16)	15
Vacation/non-work time	Time away from work either during the day or on vacation	Put[ting] my brain in a different story, in a different world, is actually a huge recharge for me. (P12)	10

##### Job control

The importance of time and calendar control were emphasized in 75% of interviews as a key to fostering vitality. A leader (P2) shared having “white space on my calendar and not over committing” is a key to a day with high vitality. This sentiment was repeated by participants explaining that vitality was highest when there was sufficient space for thinking, planning, and preparing. “The days that I have the most vitality is when I have created the space, or the space has been created throughout the day” (P16).

##### Non-work time

A key theme that emerged was taking time away from work both daily and during “vacation” time. One leader (P7) emphasized, “It’s okay to take a break. And yes, you can take a day off.” Other leaders discussed the importance of the weekend as a time for replenishment, “Trying to disconnect from work on the weekends actually brings me energy” (P9). Creating the space for vacation time was also discussed as a key to vitality.

Given the above discussion highlighting leaders’ vitality, the final research question that this study aimed to answer was how leaders effectively utilize vitality for their followers.

### Leadership behaviors

To explore this theme in greater detail, open ended questions were asked detailed in Table 4. Clear and opposing themes emerged highlighting leaders’ behaviors with high and low vitality (see Table 10).

**Table 10:** Leadership behaviors.

Participant count	Definition	Theme when high in vitality		Theme when drained of vitality	Definition	Participant count
13	Positive relational energy to others	Positive relational Energy	*VS*	Negative relational Energy	Negative relational energy to others	8
7	Active listening with a focus on curiosity, asking good questions. Being patient and focusing on the development of others	curious		closed	More shortness in communication, less curious with others, less patient, and not able to actively listen	12
7	Creating a positive environment through expressing positive emotions, being fun, happy, passionate, humorous, expressing gratitude, and using engaging body language	Positive environment		Negative environment	Creating a negative environment through being irritable, visibly stressed, not expressing gratitude, being sarcastic, showing negative emotion, and showing negative body language	9
7	A focus on encouraging others	Encouraging		Discouraging	Less capacity for emotional intelligence for others, less careful in communication, and less tolerance for mistakes.	5
6	Engaged and present with others	Engaged		Disengaged	Higher disengaged, overall lack of enjoyment of the job, and much quieter/isolated	7
6	Clear Headed, increased capacity, ambitious, and focused	High Capacity		Low Capacity	Delayed decision making, less work capacity, and more distracted	4
5	An ability to think more broadly, creatively, and focus on the big picture. More visionary leadership that creates inspiration and motivation	Visionary		Myopic	Lack of visionary leadership, less creative, and loss of being able to see the broader perspective	3
4	More collaborative, open and inclusive	Inclusive		Exclusive	Less inclusive and collaborative. Less flexible, and more inconsistent in leadership	3

#### Relational energy

The number one way that leaders expressed using their vitality was for positive relational energy. Sixty-five percent of leaders shared that when they were high in vitality, they readily shared their energy. A leader (P4) explained:

It’s contagious, right. And it’s a big part of what has allowed me to build great teams, and benefit from being a part of great teams, is being able to bring that that energy, to be a spark, to be a source of strength and confidence and influence, it’s rewarding for me, and I think it creates a great environment within which people like to work.

The same leader also recognized that the opposite was true when he felt drained of his vitality:

I tend to show it so people who know me will see it. Then it creates a different set of challenges, because they begin to wonder, is there more here? Do I have something I need to worry about? And so it ends up having a ripple effect.

Not as many leaders (40%) realized the impact of negative energy transference. Another interesting observation was that people who self-identified as extroverts shared that their positive relational energy transference felt automatic and was not a conscious choice. However, leaders that self-identified as introverts shared that transferring energy was more of an intentional choice. As one introverted leader (P12) shared, “When I am highly vital, that is when I turn externally because I have all the energy that I need. I am truly fully charged and so I seek others out.”

#### Curiosity

Leaders’ vitality also impacted their curiosity and communication. Thirty-five percent of the leaders explained that when they were high in vitality, they were able to listen to people more actively, ask good questions, were patient in conversations, and focused more on the development of others. Leader (P15) shared, “I am at my best as a CEO. I’m rarely giving answers. I’m rarely solving problems. I’m just listening.” Leaders recognized that the opposite was true when they felt drained of vitality – they become more closed off, short and abrupt in their communication, less curious of others, lacked patience and were unable to listen actively to others. A participant (P9) explained:

When I’m emotionally drained or spent it’s just much more of, just the facts. Let’s do what we need to get through this discussion or this meeting. And there’s just a whole different level of energy that I bring to the discussion.

Leaders identified how their facial expression and body language changed based on their vitality levels and how that impacted the environment around them. “It’s easy for people to pick up that something’s not quite right, because my facial expressions change, and my body language changes” (P15).

#### Environment

Participants (7 out of 20) in the study explained that when feeling high in vitality they created positive, fun environments for others through expressing positive emotions like happiness, passion, gratitude, humor, and used more engaged body language. One leader (P14) explained he was, “Very energetic, enthusiastic, optimistic, contagiously spirited. I know that I can influence if I’m in a good mood, I can influence those around me to be in a good mood.”

Leaders were also aware of the negative environment they create when they are feeling drained. Participants shared that they are more irritable, visibly stressed, lack gratitude, use sarcasm, express more negative emotion, and show their drain in their facial expression and body language. One participant (P4) shared, “I was in a meeting the other day and was irritated by something and I was just holding my head.” Environments were also impacted by their levels of encouragement to others.

#### Encouragement

Leaders acknowledged that there was a clear difference in the amount of encouragement they gave others when they were feeling vital. A leader (P5) discussed that when high in vitality he focused on encouraging the people around him, “I use the energy to point out people’s superpowers, what they are great at.” This sentiment was echoed by several (P7) of the leaders. Drains in vitality lead to an overall lack of awareness and emotional intelligence for others. Leaders shared they were less careful in their word choice and less tolerant of others including any mistakes. One leader (P12) explained she has:

No extra space for EQ, even if you intellectually know it’s there, I don’t have time. I don’t have time for your feelings. I’ve got way too many of them on my own and I’m just too tired and can’t you all see that we just need to get things done.

Given the ripple effect of leaders’ behaviors, a leader’s lack of emotional intelligence can leave followers feeling discouraged and potentially disengaged.

#### Engagement

A recognition of varying engagement levels was also identified with half of the leaders discussing the differences. Leaders shared that when they felt vital, they had a higher sense of engagement and presence with others. One participant (P1) noted that when he was feeling vital, he was, “engaged. And when I’m engaged, really engaged, that’s going to probably bring out the best of me.” When leaders felt drained, they felt disengaged, an overall lack of enjoyment of their job and they tended to be quieter or more isolated from others.

#### Capacity

Leaders acknowledged that vitality had a direct relationship with their mental capacity, with high vitality supporting being clear headed, focused, and ambitious; in contrast, drained vitality often contributed to delayed decision making, less ability to get work accomplished, and being more distracted. A participant (P4) explained, “Being clear headed comes with being fully charged.” Another leader (P10) shared the other side of the equation:

I think what happens with me is I feel like I don’t have the focus that I need to make sound decisions, I tend to also, take longer to make a decision, just because I just feel that I haven’t really done my work on something. And then sometimes you lose your motivation.

It is not surprising from this finding that leaders also felt differences in their visionary leadership based on their vitality levels.

#### Vision

Thirty percent of leaders shared that vitality was a direct contributor to how visionary their leadership was. When high in vitality, leaders shared that they had the ability to create vision, think more broadly about the organization, be more creative, and focus on the big picture, which led to more inspiration and motivation of others. One leader (P5) expressed how he uses his vitality for visionary leadership:

And then all of those things enabled me to create a vision that people believe, or they say, I think he’s the right guy to follow. It creates this, emotional connection to the vision and what we’re trying to do as a team that’s beyond what’s on the sheet of paper.

When drained of vitality, leaders shared that they lacked visionary leadership, were less creative, and lost the ability to see the broader perspective, “You lose the broader perspective that keeps things balanced” (P3).

#### Inclusion

Vitality also had an impact on whether leaders included or excluded others around them. Leaders explained that when they were high in vitality, they tended to be more collaborative, open, and inclusive. One leader (P16) explained that she had the energy to adjust her leadership style based on the needs of the different groups she was working with. She also shared that “I am more open minded to the team’s feedback and suggestions.” Leaders noted that when they felt drained of vitality that they were less inclusive, less collaborative, and less consistent in their leadership. A leader (P12) explained “It’s the time I am the least flexible. And in some ways, it’s probably the least inclusive version of me and the heaviest handed.”

This study shows that vitality is essential for both individual leadership abilities and the positive impact on followers. The leaders interviewed were able to easily recall days in which they felt highly vital and the impact that had on their followers. The participants were also highly aware of how they showed up differently when drained. Leaders recognized that being high in vitality allowed them to show up as their best self as one participant (P3) explained, “I feel it’s my times when I am at my best. There’s energy flowing out of me into vessels of people around me.”

## Discussion

Vitality is a key to combat burnout, maximize leadership capacity, and help leaders care for themselves with organizational support. This study contributes to the research base in leader vitality and is an important step in understanding how to protect leaders from burnout.

Insights from this study shed light on the psychological mechanisms that leaders are aware drain their vitality including emotional labor, self-control, and energy transference, and shows that leaders are mostly unaware of the drain of emotional dissonance. By bringing more top of mind awareness to the negative impacts of emotional dissonance, there is an opportunity to help leaders align their inner and outer emotional worlds to build stronger, more authentic relationships. Leaders must be educated on the multiple mechanisms that drain vitality to illuminate the importance of continually fostering their resources.

Additionally, there are differences in vitality drains from energy transference that potentially correlate with the personality traits of extroversion and introversion. Leaders with more extroverted personalities feel less depleted and more energized from sharing positive relational energy, where more introverted leaders feel depleted, which aligns with personality research (Cherry, 2019). Engin Deniz and Ahmet Satici (2017) examined the Big Five personality traits related to subjective vitality and found that openness, conscientiousness, extraversion, and agreeableness were all positively associated with subjective vitality, with extraversion being the most significant predictor. Perhaps at the heart of Engin Deniz and Ahmet Satici’s (2017) research finding is the relationship between extraversion and the renewable effects of positive relational energy. Bakker et al. (2006) also showed that extroverts tend to engage in intense personal interactions that counteract burnout, which can be attributed to gains in vitality.

Additional insights into vitality drains include loss of job control, the unproductive mindsets of others, and isolation created from the role. We often do not think that CEOs perceive low job control, but this study found otherwise. Support for why leaders feel a drain from loss of control comes from a meta-analytical study conducted by Park et al. (2014), which showed that lack of job control is significantly correlated with the factors of burnout, which was further supported by research in 2023 showing the effects of the Pandemic in exacerbating the issue (Chan et al., 2023). Interestingly, on the side of fostering vitality, we also see the opposite theme emerge in job autonomy. CEOs shared that having space in their day for thinking, planning, and organizing is essential in fostering their vitality. A key takeaway is to bring awareness to the importance of daily calendar and task management to foster vitality and maximize leadership capacity.

Another insight was the impact of other’s unproductive mindsets in draining vitality. People with negative, fixed, and self-focused mindsets were named as significant factors in vitality drains. This finding is consistent with research from Dutton (2003, 2014) which explains that low quality connections take an energetic toll on people, leaving them feeling depleted. Further support comes from Ebner (2022) where they completed a qualitative study examining behaviors of what they termed “energy vampires” and determined that these behaviors included: criticizing others, glorifying themselves, inflexibility in thinking, no appreciation for others, superficial or inauthentic behaviors, seeing mostly obstacles, creating problems, primarily serious in their demeanor, and lack empathy for others. CEOs and other leaders would serve themselves and the organization to recognize these behaviors and set about removing them.

Finally, the loneliness and isolation of the CEO role emerged as a theme that drains vitality. *Harvard Business Review*’s first CEO Snapshot Survey in 2012 found that half of CEOs report experiencing feelings of loneliness in their role, and of this group, 61 percent thought it hindered their performance (Harvard Business Review, 2012). CEO loneliness is a pervasive issue that was highlighted in this study (Forbes, 2020). Given that so many of the drains highlighted in this study are unchangeable demands of the role, it is essential that leaders understand how to foster their vitality.

Several of the PERMA+4 pathways were highlighted as keys to fostering vitality with relationships as the most mentioned theme followed by physical health, accomplishment, mindset, meaning, environment, and engagement. This study’s findings on the importance of relationships are consistent with research in High Quality Connections (HQC) which are characterized by an uplifted feeling a person gets through a positive interaction at work (Spreitzer et al., 2011; Waters et al., 2022). We see from both the drains and the fosters of vitality that relationships matter, but more importantly quality relationships matter.

Another critical theme that emerged was physical health. Although many leaders know that physical health is important to vitality, leaders often sacrifice their health due to the high demands of their roles. Unfortunately, top level leaders are often deprioritizing physical health, absorbing high levels of stress, traveling extensively, sleeping less, and consuming unhealthy diets, leading to 50% higher costs for health-related illnesses over other level employees (Flemming, 2021). This study provides further evidence that there is a significant relationship between physical health and vitality; however, it is not enough just knowing this connection – leaders need to focus on prioritizing their health and organizations need to support them.

Two other themes emerged including job autonomy and the importance of time away from work. We see that job autonomy, even at the CEO level, is critical to fostering vitality. Support for this finding comes from Tummers et al.’s (2018) finding that employees’ subjective vitality was positively impacted by job autonomy, and from Hätinen et al.’s (2007) results that perceived job control mediated decreases in burnout. This study highlights that even at the CEO level, leaders are not exercising control over their calendars enough to create the daily vitality needed and therefore should take back control.

This study further supports that CEOs and all leaders need to care for themselves in order to care for others including taking time away from work. Although this insight seems obvious, time away from works continues to be a struggle at both top leadership levels and throughout organizations. A study completed in January of 2022 showed that on average, employees in the U.S. had 9.5 unused vacation days left at the end of 2021 (Qualtrics, 2022). CEOs not taking vacation time is a well-documented issue with several articles highlighting this issue; for example, the CEO of Whole Foods had $613,836 banked because of 2,703 time-off hours he had not used in his 24 years at the company (Chicago Tribune, 2015). This study highlighted that not only is vacation time essential, having daily time away from work is also critical, but challenging. As one leader (P12) shared, “If I am not reading, it is a big red flag. And ironically, I have not picked up a book in probably three months.” Leaders at every level should be aware of the important vitality gains from time away from work and create the needed boundaries to make it happen on a continuous basis.

One of the most interesting insights of the study is how leaders utilize their vitality for their followers and the potential negative consequences to their leadership when drained. This study further underscores the importance of vitality as a key to impacting others. Eight opposing themes emerged around how leaders utilize their vitality for followers. Leaders high in vitality use that positive energy to transfer it to others, create positive and inclusive environments, engage with others, encourage the people around them, show up with curiosity, and overall have more mental capacity for their roles. On the other side, leaders that are drained transfer negative energy, create environments that are negative and exclusive, show up closed and discouraging to others, and have lower mental capacity. Leaders need to care for their vitality not only as a protection against burnout, but also as a key to maximizing their leadership performance.

This study also showed that the drains, fosters, and uses of leader vitality are more universal and may not be directly influenced by demographic differences. The sample for this study was intentionally selected to include gender and ethnic diversity as well as cut across multiple industries. Interestingly, there were no significant differences in themes based on demographic or industry factors. This homogeneity shows that leader vitality may be a general concept at the CEO level and potentially more influenced by personality traits. However, more research should be done with larger sample sizes to confirm this finding.

This study provides insight into leader vitality from the perspective of some of the most influential and pressured leaders in the country. The hope is that this knowledge can now be applied to a broader leadership audience and make a significant contribution to how organizations support leaders in the future with a focus on leader vitality.

### Limitations and future research directions

There are several limitations of this study that could be addressed with future research. First, this study focused on leaders only in the US, limiting the generalizability of the results. Also, the qualitative study is an extreme sample from the top level of US based organizations. Future research should include international participants and leaders at multiple levels. Another limitation of this study is related to diversity. This study used convenience sampling, which limits the diversity of participants. It is important to study leader vitality and well-being in larger and more diverse samples across multiple countries. This study was also cross-sectional in nature which makes the findings limited. Future longitudinal research could help us better understand leader vitality over extended periods. Future research can further explore the early findings in personality dimensions for vitality and should explore the Big Five factors of personality. Finally, given the unexpected absence of positive emotions as a theme in fostering vitality, additional studies can explore this finding.

## Conclusion

This study helps to further the academic research in leader vitality by providing a summary of what drains and fosters this important resource. The importance of vitality as a foundation to leadership was clear from the results: leaders need to care for themselves to care for others. “You cannot serve from an empty vessel” (Brownn, 2020). This study serves as a contribution to the research in leader vitality to support leaders and organizations in protecting leaders from burnout, maximizing leadership capacity, and helping leaders care for themselves with organizational support.

## Data availability statement

The original contributions presented in the study are included in the article/Supplementary material, further inquiries can be directed to the corresponding author.

## Ethics statement

The studies involving humans were approved by Claremont Graduate University IRB. The studies were conducted in accordance with the local legislation and institutional requirements. The participants provided their written informed consent to participate in this study.

## Author contributions

The author confirms being the sole contributor of this work and has approved it for publication.

## References

[ref1] Abraham R. (1998). Emotional dissonance in organizations: antecedents, consequences, and moderators. Genet. Soc. Gen. Psychol. Monogr. 124, 229–246. PMID: 9597747

[ref2] Abraham R. (1999). The impact of emotional dissonance on organizational commitment and intention to turnover. J. Psychol. 133, 441–455. doi: 10.1080/00223989909599754, PMID: 10412221

[ref3] Abubakar A. M. Rezapouraghdam H. Behravesh E. Megeirhi H. A. (2022). Burnout or boreout: a meta-analytic review and synthesis of burnout and boreout literature in hospitality and tourism. J. Hosp. Market. Manag. 31, 458–503. doi: 10.1080/19368623.2022.1996304

[ref4] Andela M. Truchot D. (2017). Emotional dissonance and burnout: the moderating role of team reflexivity and re-evaluation. Stress. Health 33, 179–189. doi: 10.1002/smi.2695, PMID: 27430866

[ref5] Andela M. Truchot D. Van der Doef M. (2016). Job stressors and burnout in hospitals: the mediating role of emotional dissonance. Int. J. Stress. Manag. 23, 298–317. doi: 10.1037/str0000013

[ref6] Avolio B. J. Gardner W. L. Walumbwa F. O. Luthans F. May D. R. (2004). Unlocking the mask: a look at the process by which authentic leaders impact follower attitudes and behaviors. Leadersh. Q. 15, 801–823. doi: 10.1016/j.leaqua.2004.09.003

[ref8] Bakker A. B. Demerouti E. Sanz-Vergel A. I. (2014). Burnout and work engagement: the jd–r approach. Annu. Rev. Organ. Psych. Organ. Behav. 1, 389–411. doi: 10.1146/annurev-orgpsych-031413-091235

[ref9] Bakker A. B. Van Der Zee K. I. Lewig K. A. Dollard M. F. (2006). The relationship between the big five personality factors and burnout: a study among volunteer counselors. J. Soc. Psychol. 146, 31–50. doi: 10.3200/SOCP.146.1.31-50 16480120

[ref10] Banerjee P. Srivastava M. (2019). A review of emotional contagion. J. Manag. Res. 19, 250–266.

[ref11] Baumeister R. F. Alquist J. L. (2009). Is there a downside to good self-control? Self Identity 8, 115–130. doi: 10.1080/15298860802501474

[ref12] Brotheridge C. M. Lee R. T. (2002). Testing a conservation of resources model of the dynamics of emotional labor. J. Occup. Health Psychol. 7, 57–67. doi: 10.1037/1076-8998.7.1.57, PMID: 11827234

[ref13] Brownn E. (2020) Available at: http://www.eleanorbrownn.com/

[ref14] Cameron K. S. (2021). Positively energizing leadership: Virtuous actions and relationships that create high performance. San Francisco: Berrett-Koehler Publishers.

[ref15] Cameron K. Mora C. Leutscher T. Calarco M. (2011). Effects of positive practices on organizational effectiveness. J. Appl. Behav. Sci. 47, 266–308. doi: 10.1177/0021886310395514

[ref16] Cannon M. (2011). Do leaders really need to be tired? A sustainable view of leadership development and the vital leader. Ind. Commer. Train. 43, 307–313. doi: 10.1108/00197851111145907

[ref17] Chan X. W. Shang S. Brough P. Wilkinson A. Lu C. Q. (2023). Work, life and COVID-19: a rapid review and practical recommendations for the post-pandemic workplace. Asia Pac. J. Hum. Resour. 61, 257–276. doi: 10.1111/1744-7941.12355

[ref18] Cherry K. (2019). The big five personality traits. Very Well Mind. 23:2020.

[ref19] Chicago Tribune. (2015). The story of modern CEOs and their soaring piles of unused vacation time. Available at: https://www.chicagotribune.com/business/ct-ceos-unused-vacation-time-20151229-story.html

[ref20] Crawford E. R. Lepine J. A. Rich B. L. (2010). Linking job demands and resources to employee engagement and burnout: a theoretical extension and meta-analytic test. J. Appl. Psychol. 95, 834–848. doi: 10.1037/a0019364 20836586

[ref21] Creswell J. W. Hanson W. E. Plano Clark V. L. Morales A. (2007). Qualitative research designs: selection and implementation. Counsel. Psychol. 35, 236–264. doi: 10.1177/0011000006287390

[ref22] Creswell J. W. Poth C. N. (2018). Qualitative inquiry & research design: Choosing among five approaches (fourth). London: SAGE.

[ref23] Cross R. Baker W. Parker A. (2003). What creates energy in organizations? MIT Sloan Manag. Rev. 44, 51–57.

[ref24] Deng N. Guyer R. Ware J. E. (2015). Energy, fatigue, or both? A bifactor modeling approach to the conceptualization and measurement of vitality. Qual. Life Res. 24, 81–93. doi: 10.1007/s11136-014-0839-9 25362259

[ref25] Diener E. (2009). Assessing subjective well-being: Progress and opportunities. Soc. Indic. Res. 31, 103–157. doi: 10.1007/978-90-481-2354-4

[ref26] Diestel S. Schmidt K. -H. (2009). Mediator and moderator effects of demands on self-control in the relationship between work load and indicators of job strain. Work Stress. 23, 60–79. doi: 10.1080/02678370902846686

[ref9006] Diestel S. Schmidt K. H. (2011). The moderating role of cognitive control deficits in the link from emotional dissonance to burnout symptoms and absenteeism. Journal of Occupational Health Psychology, 16, 313. 21728438 10.1037/a0022934

[ref27] Donaldson S. I. Csikszentmihalyi M. Nakamura J. (2020). Positive psychological science: Improving everyday life, well-being, work, education, and societies across the globe. London: Routledge.

[ref29] Dubreuil P. Forest J. Courcy F. (2014). From strengths use to work performance: the role of harmonious passion, subjective vitality, and concentration. J. Posit. Psychol. 9, 335–349. doi: 10.1080/17439760.2014.898318

[ref30] Dutton J. E. (2003). Energize your workplace: How to create and sustain high-quality connections at work 1st Hoboken: Jossey-Bass.

[ref31] Dutton J. E. (2014). How to be a positive leader through building high quality connections. Michigan School of Business. Center for Positive Organizations 10, 2014.

[ref32] Ebner M. (2022) Why positive leadership is a good remedy against energy vampires. Available at: https://www.linkedin.com/pulse/why-positive-leadership-good-remedy-against-energy-vampires-ebner/?trackingId=46VWGqQySlGjaj9JPVL4Bw%3D%3D

[ref33] Engin Deniz M. Ahmet Satici S. (2017). The relationships between big five personality traits and subjective vitality. Anales De Psicología 33, 218–224. doi: 10.6018/analesps.33.2.261911

[ref34] Erickson R. J. (1991). When emotion is the product: Self, society, and (in) authenticity in a postmodern world Doctoral dissertation Washington State University.

[ref35] Erickson R. J. Ritter C. (2001). Emotional labor, burnout, and inauthenticity: does gender matter? Soc. Psychol. Q. 64, 146–163. doi: 10.2307/3090130

[ref36] Flemming G. (2021). Statistics of executive burnout and derailment. Available at: https://kevinflemingphd.com/blog/articles/statistics-of-executive-burnout-and-derailment/

[ref37] Forbes (2020). Lonely at the top: how CEOs can practice conscious leadership to stop feeling isolated. Available at: https://www.forbes.com/sites/forbesbusinesscouncil/2020/05/05/lonely-at-the-top-how-ceos-can-practice-conscious-leadership-to-stop-feeling-isolated/?sh=6d888beb6bf1

[ref38] Gaddis B. Connelly S. Mumford M. D. (2004). Failure feedback as an affective event: influences of leader affect on subordinate attitudes and performance. Leadersh. Q. 15, 663–686. doi: 10.1016/j.leaqua.2004.05.011

[ref39] Gardner W. L. Avolio B. J. Luthans F. May D. R. Walumbwa F. (2005). “Can you see the real me?” a self-based model of authentic leader and follower development. Leadersh. Q. 16, 343–372. doi: 10.1016/j.leaqua.2005.03.003

[ref40] Gardner W. L. Carlson J. D. (2015). “Authentic leadership” in Wright (editor-in-chief, international encyclopedia of the Social & Behavioral Sciences). ed. James D., vol. 2. 2nd edn (Oxford: Elsevier).

[ref41] Gardner W. L. Cogliser C. C. Davis K. M. Dickens M. P. (2011). Authentic leadership: a review of the literature and research agenda. Leadersh. Q. 22, 1120–1145. doi: 10.1016/j.leaqua.2011.09.007

[ref42] Gardner W. L. Fischer D. Hunt J. G. (2009). Emotional labor and leadership: a threat to authenticity? Leadersh. Q. 20, 466–482. doi: 10.1016/j.leaqua.2009.03.011

[ref43] Garton E. (2017). Employee burnout is a problem with the company, not the person. Harv. Bus. Rev. Available at: https://hbr.org/2017/04/employee-burnout-is-a-problem-with-the-company-not-the-person

[ref44] George J. M. (1995). Leader positive mood and group performance: the case of customer service. J. Appl. Soc. Psychol. 25, 778–794. doi: 10.1111/j.1559-1816.1995.tb01775.x

[ref45] George J. M. (2000). Emotions and leadership: the role of emotional intelligence. Hum. Relat. 53, 1027–1055. doi: 10.1177/0018726700538001

[ref46] George J. M. Bettenhausen K. (1990). Understanding prosocial behavior, sales performance, and turnover: a group-level analysis in a service context. J. Appl. Psychol. 75, 698–709. doi: 10.1037/0021-9010.75.6.698

[ref47] Giampetro-Meyer A. Brown T. (1998). Working on the wiring: preventing ethical failures in socially responsible businesses. Rev. Bus. 20:8.

[ref48] Glomb T. M. Tews M. J. (2004). Emotional labor: a conceptualization and scale development. J. Vocat. Behav. 64, 1–23. doi: 10.1016/S0001-8791(03)00038-1

[ref49] Grandey A. A. (2003). When “the show must go on”: surface acting and deep acting as determinants of emotional exhaustion and peer-rated service delivery. Acad. Manag. J. 46, 86–96. doi: 10.2307/30040678

[ref50] Grandey A. A. Kern J. H. Frone M. R. (2007). Verbal abuse from outsiders versus insiders: comparing frequency, impact on emotional exhaustion, and the role of emotional labor. J. Occup. Health Psychol. 12, 63–79. doi: 10.1037/1076-8998.12.1.63, PMID: 17257067

[ref51] Greenglass E. R. (2006). Vitality and vigor: Implications for healthy functioning. Stress and anxiety: application to health, work place, community and education. Tyne: Cambridge Scholars Press.

[ref52] Harvard Business Review (2012). It’s time to acknowledge CEO loneliness. Available at: https://hbr.org/2012/02/its-time-to-acknowledge-ceo-lo

[ref53] Hätinen M. Kinnunen U. Pekkonen M. Kalimo R. (2007). Comparing two burnout interventions: perceived job control mediates decreases in burnout. Int. J. Stress. Manag. 14, 227–248. doi: 10.1037/1072-5245.14.3.227

[ref54] Hennink M. Kaiser B. N. (2022). Sample sizes for saturation in qualitative research: a systematic review of empirical tests. Soc. Sci. Med. 292:114523. 34785096 10.1016/j.socscimed.2021.114523

[ref55] Hoch J. E. Bommer W. H. Dulebohn J. H. Wu D. (2018). Do ethical, authentic, and servant leadership explain variance above and beyond transformational leadership? A meta- analysis. J. Manage. 44, 501–529. doi: 10.1177/0149206316665461

[ref56] HR Executive (2021). Number of the day: leader burnout. Available at: https://hrexecutive.com/number-of-the-day-leader-burnout/

[ref57] Hsieh H. Shannon S. E. (2005). Three approaches to qualitative content analysis. Qual. Health Res. 15, 1277–1288. doi: 10.1177/1049732305276687 16204405

[ref58] Humphrey R. H. (2002). The many faces of emotional leadership. Leadersh. Q. 13, 493–504. doi: 10.1016/S1048-9843(02)00140-6

[ref59] Humphrey R. H. Ashforth B. E. Diefendorff J. M. (2015). The bright side of emotional labor: the bright side of emotional labor. J. Organ. Behav. 36, 749–769. doi: 10.1002/job.2019

[ref60] Humphrey R. H. Pollack J. M. Hawver T. (2008). Leading with emotional labor. J. Manag. Psychol. 23, 151–168. doi: 10.1108/02683940810850790

[ref61] Ilies R. Morgeson F. P. Nahrgang J. D. (2005). Authentic leadership and eudaemonic well-being: understanding leader-follower outcomes. Leadersh. Quar. 16, 373–394. doi: 10.1016/j.leaqua.2005.03.002

[ref62] Johnson S. K. (2008). I second that emotion: effects of emotional contagion and affect at work on leader and follower outcomes. Leadersh. Q. 19, 1–19. doi: 10.1016/j.leaqua.2007.12.001

[ref63] Johnson H. -A. M. Spector P. E. (2007). Service with a smile: do emotional intelligence, gender, and autonomy moderate the emotional labor process? J. Occup. Health Psychol. 12, 319–333. doi: 10.1037/1076-8998.12.4.319, PMID: 17953492

[ref64] Ju H. (2017). The relationship between physical activity, meaning in life, and subjective vitality in community-dwelling older adults. Arch. Gerontol. Geriatr. 73, 120–124. doi: 10.1016/j.archger.2017.08.001, PMID: 28802214

[ref65] Kenworthy J. Fay C. Frame M. Petree R. (2014). A meta-analytic review of the relationship between emotional dissonance and emotional exhaustion. J. Appl. Soc. Psychol. 44, 94–105. doi: 10.1111/jasp.12211

[ref66] Kiker D. S. Callahan J. S. Kiker M. B. (2019). Exploring the boundaries of servant leadership: a meta-analysis of the main and moderating effects of servant leadership on behavioral and affective outcomes. J. Manager. Issues 31, 172–197.

[ref68] Kondracki N. L. Wellman N. S. (2002). Content analysis: review of methods and their applications in nutrition education. J. Nutr. Educ. Behav. 34, 224–230. doi: 10.1016/S1499-4046(06)60097-3 12217266

[ref69] Lavrusheva O. (2020). The concept of vitality. Review of the vitality-related research domain. New Ideas Psychol. 56:100752. doi: 10.1016/j.newideapsych.2019.100752

[ref70] Lee J. S. Oh W. O. (2007). Factors influencing vitality among nurses. J. Korean Acad. Nurs. 37, 676–683. doi: 10.4040/jkan.2007.37.5.676, PMID: 17804934

[ref71] Lewis K. M. (2000). When leaders display emotion: how followers respond to negative emotional expression of male and female leaders. J. Organ. Behav. 21, 221–234. doi: 10.1002/(SICI)1099-1379(200003)21:2<221::AID-JOB36>3.0.CO;2-0

[ref72] Li Y. N. Law K. S. Yan M. (2019). Other-caring or other-critical? A contagious effect of leaders’ emotional triads on subordinates’ performance. Asia Pac. J. Manag. 36, 995–1021. doi: 10.1007/s10490-018-9617-5

[ref73] Liao C. Lee H. W. Johnson R. E. Lin S. H. (2021). Serving you depletes me? A leader-centric examination of servant leadership behaviors. J. Manag. 47, 1185–1218. doi: 10.1177/0149206320906883

[ref9007] Martínez-Iñigo D. Totterdell P. Alcover C. M. Holman D. (2007). Emotional labour and emotional exhaustion: Interpersonal and intrapersonal mechanisms. Work & Stress, 21, 24330–47.

[ref74] Maxwell J. A. (2004). Using qualitative methods for causal explanation. Field Methods 16, 243–264. doi: 10.1177/1525822X04266831

[ref75] McNair D. M. (1971). Profile of mood states instrument. Manu. Profile Mood States, 3–29.

[ref76] Middleton D. R. (1989). Emotional style: the cultural ordering of emotions. Ethos 17, 187–201. doi: 10.1525/eth.1989.17.2.02a00030

[ref77] Morris J. A. Feldman D. C. (1996). The dimensions, antecedents, and consequences of emotional labor. Acad. Manag. Rev. 21, 986–1010. doi: 10.2307/259161

[ref78] Muraven M. Shmueli D. Burkley E. (2006). Conserving self-control strength. J. Pers. Soc. Psychol. 91, 524–537. doi: 10.1037/0022-3514.91.3.524 16938035

[ref79] O’Brien E. Linehan C. (2019). Problematizing the authentic self in conceptualizations of emotional dissonance. Hum. Relat. 72, 1530–1556. doi: 10.1177/0018726718809166

[ref80] Owens B. P. Baker W. E. Sumpter D. M. D. Cameron K. S. (2016). Relational energy at work: implications for job engagement and job performance. J. Appl. Psychol. 101, 35–49. doi: 10.1037/apl0000032 26098165

[ref9008] Penninx B. W. Guralnik J. M. Simonsick E. M. Kasper J. D. Ferrucci L. Fried L. P. (1998). Emotional vitality among disabled older women: The Women’s Health and Aging Study. Journal of the American Geriatrics Society, 46, 807–815. 9670865 10.1111/j.1532-5415.1998.tb02712.x

[ref81] Park H. I. Jacob A. C. Wagner S. H. Baiden M. (2014). Job control and burnout: a meta-analytic test of the conservation of resources model. Appl. Psychol. 63, 607–642. doi: 10.1111/apps.12008

[ref82] Penninx B. W. J. H. Guralnik J. M. Bandeen-Roche K. Kasper J. D. Simonsick E. M. Ferrucci L.. (2000). The protective effect of emotional vitality on adverse health outcomes in disabled older women. J. Am. Geriatr. Soc. 48, 1359–1366. doi: 10.1111/j.1532-5415.2000.tb02622.x, PMID: 11083309

[ref83] Perlman B. Hartman E. A. (1982). Burnout: summary and future research. Hum. Relat. 35, 283–305. doi: 10.1177/001872678203500402

[ref84] Qualtrics. (2022). Half of U.S. employees say they WFV: Work From Vacation. Available at: https://www.qualtrics.com/news/half-of-u-s-employees-say-they-wfv-work-from-vacation/

[ref85] Richman L. S. Kubzansky L. D. Maselko J. Ackerson L. K. Bauer M. (2009). The relationship between mental vitality and cardiovascular health. Psychol. Health 24, 919–932. doi: 10.1080/08870440802108926 20205036

[ref86] Rivkin W. Diestel S. Schmidt K.-H. (2015). Affective commitment as a moderator of the adverse relationships between day-specific self-control demands and psychological well-being. J. Vocat. Behav. 88, 185–194. doi: 10.1016/j.jvb.2015.03.005

[ref87] Rod Laird Organisation (2012). Extreme or deviant case sampling: An essential way to learn from both success and failure. London: Rods Reflections.

[ref88] Rubin R. S. Tardino V. M. S. Daus C. S. Munz D. C. (2005). A reconceptualization of the emotional labor construct: on the development of an integrated theory of perceived emotional dissonance and emotional labor. In Annual conference of the Society for Industrial and Organizational Psychology, 17th, Toronto, ON, Canada A version of this chapter was presented at the aforementioned conference. Lawrence Erlbaum Associates Publishers.

[ref89] Ryan R. Deci E. (2008). From ego depletion to vitality: theory and findings concerning the facilitation of energy available to the self. Soc. Personal. Psychol. Compass 2, 702–717. doi: 10.1111/j.1751-9004.2008.00098.x

[ref91] Ryan R. M. Frederick C. (1997). On energy, personality, and health: subjective vitality as a dynamic reflection of well-being. J. Pers. 65, 529–565. doi: 10.1111/j.1467-6494.1997.tb00326.x, PMID: 9327588

[ref92] Schmidt K. H. Neubach B. Heuer H. (2007). Self-control demands, cognitive control deficits, and burnout. Work Stress 21, 142–154. doi: 10.1080/02678370701431680

[ref93] Seligman M. (2018). PERMA and the building blocks of well-being. J. Posit. Psychol. 13, 333–335. doi: 10.1080/17439760.2018.1437466

[ref94] Shapiro J. Donaldson S. I. (2022). The leader vitality scale (LVS): development, psychometric assessment, and validation. Front. Psychol. 13:884672. doi: 10.3389/fpsyg.2022.884672 35756249 PMC9226326

[ref95] Spector P. E. (1998). “A control theory of the job stress process” in Theories of organizational stress. ed. Cooper C. L. (New York: Oxford University Press)

[ref96] Spreitzer G. M. Cameron K. S. Stephens J. P. Heaphy E. Dutton J. E. (2011). High-quality connections. The Oxford handbook of positive organizational scholarship. Oxford: Oxford University Press.

[ref97] Sy T. Cote S. Saavedra R. (2005). The contagious leader: impact of the leader’s mood on the mood of group members, group affective tone, and group processes. J. Appl. Psychol. 90, 295–305. doi: 10.1037/0021-9010.90.2.295, PMID: 15769239

[ref98] Tangney J. P. Baumeister R. F. Boone A. L. (2004). High self-control predicts good adjustment, less pathology, better grades, and interpersonal success. J. Pers. 72, 271–324. doi: 10.1111/j.0022-3506.2004.00263.x, PMID: 15016066

[ref99] Thayer R. E. (1996). The origin of everyday moods: Managing energy, tension, and stress. Oxford: Oxford University Press.

[ref100] The Society Pages (2020). Fortune 500 CEOs, 2000-2020: still male, still white. Available at: https://thesocietypages.org/specials/fortune-500-ceos-2000-2020-still-male-still-white/

[ref101] Tummers L. Steijn B. Nevicka B. Heerema M. (2018). The effects of leadership and job autonomy on vitality: survey and experimental evidence. Rev. Public Pers. Admin. 38, 355–377. doi: 10.1177/0734371X16671980, PMID: 30100670 PMC6055114

[ref102] Van Dijk P. A. Kirk Brown A. (2006). Emotional labour and negative job outcomes: an evaluation of the mediating role of emotional dissonance. J. Manag. Organ. 12, 101–115. doi: 10.5172/jmo.2006.12.2.101

[ref103] Vogel B. (2017). Experiencing human energy as a catalyst for developing leadership capacity. Developing Leaders for Positive Organizing. Bingley: Emerald Publishing Limited.

[ref104] Wang G. (2011). What role does leaders’ emotional labor play in effective leadership? An empirical examination. Unpublished PhD dissertation: University of Lowa.

[ref105] Waters L. Algoe S. B. Dutton J. Emmons R. Fredrickson B. L. Heaphy E.. (2022). Positive psychology in a pandemic: buffering, bolstering, and building mental health. J. Posit. Psychol. 17, 303–323. doi: 10.1080/17439760.2021.1871945

[ref106] Watson D. Tellegen A. (1985). Toward a consensual structure of mood. Psychol. Bull. 98, 219–235. doi: 10.1037/0033-2909.98.2.219, PMID: 3901060

[ref107] Weiss M. Razinskas S. Backmann J. Hoegl M. (2018). Authentic leadership and leaders’ mental well-being: an experience sampling study. Leadersh. Q. 29, 309–321. doi: 10.1016/j.leaqua.2017.05.007

[ref108] Winkler A. D. Zapf D. Kern M. (2023). Effects of emotion-rule dissonance on emotional exhaustion and physiological health: a two-wave study. Appl. Psychol. doi: 10.1111/apps.12489

[ref109] Zapf D. Vogt C. Seifert C. Mertini H. Isic A. (1999). Emotion work as a source of stress: the concept and development of an instrument. Eur. J. Work Organ. Psy. 8, 371–400. doi: 10.1080/135943299398230

