# Supplementary material for: Burning bright or burning out: a qualitative investigation of leader vitality
Source: Front Psychol. 2023 Oct 2;14:1244089. doi: 10.3389/fpsyg.2023.1244089 (PMC10581267; doi:10.3389/fpsyg.2023.1244089)
Supplement: Supplementary file 1 [file Table_11.docx]

**Appendix A**

**Table 11**

*Final Coding Scheme*

| Leader Vitality | Code Description |
| --- | --- |
| Antecedents – Foster  Based on the PERMA+4 Model | Positive Emotions  Engagement  Positive Relationships  Meaning  Accomplishment  Health  Mindset  Environment  Economic Security  Job Control  Vacation/Non-work time |
| Drains – Based on psychological mechanisms | Self-Control  Emotional Labor  Emotional Dissonance  Energy Transference  Loss of Job Control  Unproductive Mindsets of Others  Isolation |
| Leadership Behaviors – High in Vitality | Positive Relational Energy  Curious  Positive Environment  Encouraging  Engaged  High Capacity  Visionary  Inclusive |
| Leadership Behaviors – Drained in Vitality | Negative Relational Energy  Closed  Negative Environment  Discouraging  Disengaged  Low Capacity  Myopic  Exclusive |
